# Aspergillus-specific IgM/IgG antibody serostatus of patients hospitalized with moderate-critical COVID-19 in Uganda

**DOI:** 10.4314/ahs.v22i3.54

**Published:** 2022-09

**Authors:** Felix Bongomin, Richard Kwizera, Emmanuel Mande, Sharley Melissa Aloyo, Beatrice Achan, Martha Namusobya, Senai Goitom Sereke, Charles Batte, Sarah Kiguli, Joseph Baruch Baluku, Moses L Joloba, Bruce J Kirenga

**Affiliations:** 1 Department of Medical Microbiology and Immunology, Gulu University Medical School, Gulu Uganda; 2 Translational research laboratory, Department of Research, Infectious Diseases Institute, College of Health Sciences, Makerere University, P.O Box 22418, Kampala, Uganda; 3 Department of Immunology and Molecular Biology, Makerere University College of Health Sciences, Kampala, Uganda; 4 Department of Medical Microbiology, College of Health Sciences, Makerere University, Kampala, Uganda; 5 Department of Internal Medicine, Mulago National Referral Hospital, Kampala, Uganda; 6 Department of Radiology and Radiotherapy, College of Health Sciences, Makerere University, Kampala, Uganda; 7 Makerere University Lung Institute, College of Health Sciences, Makerere University, Kampala, Uganda; 8 Department of Pediatrics and Child Health, College of Health Sciences, Makerere University, Kampala, Uganda; 9 Directorate of Programs, Mildmay Uganda, Wakiso, Uganda

**Keywords:** Invasive pulmonary aspergillosis, COVID-19, critical illness, Uganda

## Abstract

Invasive pulmonary aspergillosis is known to complicate the coronavirus diseases-2019 (COVID-19), especially those with critical illness. We investigated the baseline anti-Aspergillus antibody serostatus of patients with moderate-critical COVID-19 hospitalized at 3 COVID-19 Treatment Units in Uganda. All 46 tested patients, mean age 30, and 11% with underlying respiratory disease had a negative serum anti-Aspergillus IgM/IgG antibody immunochromatographic test on day 3 (mean) of symptom onset (range 1–26), but follow up specimens to assess seroconversion were not available.

## Background

The coronavirus disease-2019 (COVID-19), caused by the severe acute respiratory syndrome coronavirus-2 (SARS-CoV-2), has affected millions of individuals since December 2019, resulting in significant morbidity and mortality globally^1^. As of 30^th^ September, 2021, over 234 million had contracted the disease, resulting to over 4.8 million deaths, worldwide^1^. Experience from previous influenza pandemics have shown that microbial co-infection increases the risk of disease severity in humans infected with viral infections^2^. A number of studies have documented SARS-CoV-2 co-infection with other pathogens. Moreover, microbial co-infection occurs very early in patients with COVID-19^3^.

Aspergillus species are ubiquitous environmental molds that cause a spectrum of clinical pulmonary syndromes ranging from allergic, invasive and chronic disease with varying severity and clinical outcomes depending on the immune status of the host and the virulence of the etiologic Aspergillus species^4^. Recently, fungal pathogens notably Candida, Histoplasma, Cryptococcus and Aspergillus species co-infections have been reported in patients with COVID-19 associated with a particularly poor outcomes^5^,^6^.

COVID-19 associated pulmonary aspergillosis (CAPA), is frequently observed in patients with moderate to severe COVID-19 disease, especially those on immunosuppressive therapy, underlying lung disease and those requiring invasive mechanical ventilation or extracorporeal membranous oxygenation^7^,^8^. In published case series, CAPA has been reported to have a very high mortality rate in excess of 50%^7^–^9^. Therefore, early recognition and institution of appropriate antifungal agents is key to improve outcomes^8^.

As of 30^th^ September, 2021, Uganda has recorded over 123,500 cases of COVID-19 with over 3,150 of these patients dying from the disease^1^. In the first wave of the pandemic, much as a vast majority of Ugandan patients diagnosed with COVID-19 were asymptomatic or had a mild disease (10), about 4% of the patients had moderate to severe disease. However, in the second wave, close to 80% of hospitalized patients had moderate-severe COVID-19^11^. These patients are at increased risk of CAPA. This study therefore, aimed at determining anti-Aspergillus-specific IgG/IgM serostatus in patients with moderate and severe COVID – 19 in Uganda to guide future studies and clinical care of these patients who are at risk of CAPA.

## Patients and Methods

In January 2021, we conducted a laboratory-based, retrospective observational study on blood samples of patients hospitalized and managed for COVID-19 at three COVID-19 treatment units (CTUs) in Uganda, namely Mulago National Referral Hospital (MNRH), Entebbe Regional Referral Hospital (ERRH) and Arua Regional Referral Hospital (ARRH).

Biobanked serum samples of patients hospitalized with moderate, severe, critical COVID-19 defined by the need for oxygenation or mechanical ventilation were randomly selected. Demographic characteristics, co-morbidities and clinical outcomes of the patients were retrieved from the Integrated Biorepository of H3Africa Uganda (IBRH3AU) electronic database. IBRH3AU is situated in the Department of Immunology and Molecular Biology, School of Biomedical Sciences, Makerere University College of Health Sciences, Kampala, Uganda. The biorepository has in storage well annotated different sample types from over 500 COVID-19 patients and out of these, 46 serum samples of the eligible patients were randomly selected.

Thawed serum samples of the eligible patients were examined using the commercially available anti-Aspergillus IgM/IgG antibody immunochromatographic technology (ICT) lateral flow assay, which has excellent sensitivity (88.9–91.6%) and specificity (96.3–98%) (LDBio Diagnostics, Lyon, France)^12^–^15^. STATA version 16 was used to produce descriptive statistics.

## Patient Consent Statement

This study was approved by the Makerere University School of Biomedical Sciences Research and Ethics Committee Approval number; MHREC 1868. Patients records were registered using anonymous alpha-numeric codes and principles outlined in the Declaration of Helsinki were observed.

## Results

We tested 46 serum samples from 46 unique individuals (Male n=38, Female n=8). The median age of the participants was 34 (range: 14–54) years. Forty (87%) patients had co-morbidities, mainly diabetes and cardiovascular diseases. The median duration of illness prior to hospitalization was 3 days (range: 1–26 days). Shortness of breath was the most common symptom reported by 42 (91.3%) participants followed by cough (37%), headache (32.6%) and fever (26.1%). Table 1

**Table 1:** Characteristics of patients evaluated for Aspergillus IgG/IgM serostatus

Characteristics	Frequency /median	Percent/range
**Treatment center**		
Entebbe Regional Referral Hospital Mulago National Referral Hospital Arua Regional Referral Hospital	20 17 9	43.5 37.5 19.6
**Sex**		
Male Female	38 8	82.6 17.4
**Age, years**	34	14–54
**Occupation**		
Truck drivers Business Professionals Students	20 12 12 2	43.5 26.1 26.1 4.3
**Clinical symptoms**		
Fever Cough Hemoptysis Rhinorrhea Chest pain Shortness of breath Headache	12 17 1 12 4 42 15	26.1 37.0 2.2 26.1 8.7 91.3 32.6
Comorbidities	40	87.0
Diabetes mellitus alone Hypertension alone HIV alone Diabetes and hypertension Diabetes + HIV Asthma Chronic obstructive pulmonary disease alone	12 8 3 10 2 3 2	30.0 20.0 7.5 25 5 7.5 5
**Treatment**		
Azithromycin Hydroxychloroquine Dexamethasone	27 12 16	58.7 26.1 34.8
**Level of care**		
Intensive care unit –invasive mechanical ventilation Intensive care unit – non-invasive ventilation High dependency unit - Oxygenation	8 8 30	17.4 17.4 65.2

Sixteen (34.8%) patients were hospitalized in the intensive care unit, with 8 (50%) of them requiring invasive mechanical ventilation. Twenty-seven (58.7%) patients received azithromycin, 12 (26.1%) hydroxychloroquine and 16 (34.8%) dexamethasone in addition to supportive care. Table 1

Serum anti-Aspergillus IgM/IgG lateral flow assay tests were negative for all the patients.

## Discussion

There is increasing evidence on the increased frequency and poor outcomes of invasive pulmonary aspergillosis in patients with severe/critical COVID-19^9^. In this report, we investigated baseline anti-Aspergillus antibody serostatus of patients hospitalized with moderate-critical COVID-19 in Uganda. All the participants tested negative for serum anti-Aspergillus IgM/IgG antibody. These findings can be interpreted in 2 ways. Firstly, CAPA may be an uncommon occurrence in our settings. This is supported by a large retrospective study reporting a relatively lower incidence of invasive aspergillosis in patients with COVID-19 acute respiratory distress syndrome (ARDS) compared with non-COVID-19 ARDS^16^. Secondly, serum anti-Aspergillus IgM/IgG lateral flow assay may be an unreliable screening tool for CAPA. This is consistent with findings from recent studies showing that conventional diagnostics for invasive pulmonary aspergillosis may miss most cases of CAPA^17^. However, this assay has been validated for the diagnosis of invasive pulmonary aspergillosis and sub-acute invasive pulmonary aspergillosis^12^. Also, the median duration of hospitalization for the participants was short, as CAPA has been reported to occur in around the 8^th^ to 10^th^ day^17^. A negative anti-Aspergillus IgM/IgG also rule out any possibility of underlying chronic pulmonary or allergic bronchopulmonary aspergillosis among the participants^13^.

The knowledge of the risk factors and burden of CAPA remains limited. There is no known established risk factor for the development of CAPA and patients with CAPA in most cases do not have the classic risk factors for invasive aspergillosis^18^,^19^. A study from France did not find any difference between patients with and without invasive pulmonary aspergillosis regarding age, gender, medical history and severity on admission and during hospitalization^20^. In this particular study, high doses of corticosteroids and azithromycin usage were associated with increased frequency of occurrence of invasive aspergillosis^20^. In published literature, the incidence of CAPA has been reported to range anywhere between 20 and 35%^19^. However, prolonged corticosteroid therapy, ICU admission and COVID-19 itself may be possible risk factor for this emerging disease (18). Based on small case series, mortality rates associated with CAPA is as high as 70%^19^,^20^.

Thus, the diagnostic work up for CAPA remains a work in progress. Among ICU patients, CAPA patients had a lower positive culture or polymerase chain reaction (PCR) or galactomannan test on bronchoalveolar lavage fluid compared to influenza patients^17^. A panel of experts on CAPA suggests that the European Organization for Research and Treatment of Cancer and the Mycosis Study Group Education and Research Consortium definitions for invasive fungal disease^21^ may not be suitable for the diagnosis of CAPA or its related syndrome - influenza-associated pulmonary aspergillosis^18^. This panel proposed CAPA to be defined as possible, probable, or proven on the basis of sample validity and thus diagnostic certainty^18^. A proven diagnosis of CAPA requires at least one of the following: histopathological or direct microscopic detection of fungal hyphae, showing invasive growth with associated tissue damage; or Aspergillus recovered by culture or microscopy or histology or PCR obtained by a sterile aspiration or biopsy from a pulmonary site, showing an infectious disease process^18^.

To-date, no randomized clinical trial has evaluated any antifungal regimen for the treatment of CAPA. Current treatment approach is based on the basic principles of antifungal therapy extrapolated from experience and evidence of treating invasive aspergillosis in other population. As such, voriconazole or isavuconazole have been recommended as first-line treatment for possible, probable, and proven CAPA^18^. Cases of azole-resistant Aspergillus fumigatus causing CAPA has been reported^22^. This poses therapeutic dilemma. Alternative antifungal agents such as amphotericin B and the echinocandins may as well have a role in the management of CAPA. However, evidence on the use of these agents remains scarce.

## Limitation

The reader should consider the following limitation while interpreting the finding of our study. Importantly, the diagnosis of CAPA requires a combination of factors including mycological, host, clinical and histopathological factors^18^,^21^,^23^. Therefore, evaluation of a patient based on a single modality is best considered a screening tool. Also, our sample size is very small and we may have missed some cases in the larger sample pool.

In summary, among patients hospitalized with severe/critical COVID-19 in Uganda, we failed to find any evidence of current or prior immune response to Aspergillus infection. This finding potentially provides a useful clinical information on the rarity of Aspergillus infection among COVID-19 patients in Uganda. However, further research utilizing better diagnostic modalities is required.

## Data Availability

The underlying data can be accessed through the corresponding author on reasonable request.
